# Comparison of methods for quantifying primordial follicles in the mouse ovary

**DOI:** 10.1186/s13048-020-00724-6

**Published:** 2020-10-14

**Authors:** Urooza C. Sarma, Amy L. Winship, Karla J. Hutt

**Affiliations:** 1 Development and Stem Cells Program, Monash Biomedicine Discovery Institute, Level 3, Building 76, 19 Innovation walk, Clayton, VIC 3800 Australia; 2 Department of Anatomy and Developmental Biology, Monash University, Level 3, Building 76, 19 Innovation walk, Clayton, VIC 3800 Australia

**Keywords:** Ovary, Follicle counting, Ovarian reserve, Counting methods, Stereology

## Abstract

**Background:**

Accurate evaluation of primordial follicle numbers in mouse ovaries is an essential endpoint for studies investigating how endogenous and exogenous insults, such as maternal aging and chemotherapy, impact the ovarian reserve. In this study, we compared and contrasted two methods for counting healthy primordial follicles following exposure to cyclophosphamide (75 mg/kg), a well-established model of follicle depletion. The first was the fractionator/optical dissector technique, an unbiased, assumption-free stereological approach for quantification of primordial follicle numbers. While accurate, highly reproducible and sensitive, this method relies on specialist microscopy equipment and software, requires specific fixation, embedding and sectioning parameters to be followed, and is largely a manual process that is tedious and time-consuming. The second method was the more widely used serial section and direct count approach, which is relatively quick and easy. We also compared the impacts of different fixatives, embedding material and section thickness on the overall results for each method.

**Results:**

Direct counts resulted in primordial follicle numbers that were significantly lower than those obtained by stereology, irrespective of fixation and embedding material. When applied to formalin fixed tissue, the direct count method did not detect differences in follicle numbers between saline and cyclophosphamide treated groups to the same degree of sensitivity as the gold standard stereology method (referred to as the Reference standard). However, when Bouin’s fixative was used, direct counts and stereology were comparable in their ability to detect follicle depletion caused by cyclophosphamide.

**Conclusions:**

This work indicates that the direct count method can produce similar results to stereology when Bouin’s fixative is used instead of formalin. The findings presented here will assist others to select the most appropriate experimental approach for accurate follicle enumeration, depending on whether the primary objective of the study is to determine absolute primordial follicle numbers or relative differences between groups.

## Introduction

The ovarian reserve of primordial follicles represents the entire stockpile of gametes available to females [1, 2]. Primordial follicles consist of a singular oocyte arrested at diplotene of meiotic prophase 1, surrounded by one layer of squamous granulosa cells [3–5]. These remain in a quiescent state until recruited to undergo a folliculogenesis, ultimately culminating in follicular atresia or ovulation of a mature oocyte [6, 7]. This process causes a slow decline in the supply of primordial follicles until exhaustion of the reserve, leading to infertility followed by menopause [2, 8, 9]. Once exhausted, there is no replenishment of the ovarian reserve [10, 11]. Therefore, the length of the female fertile lifespan, from puberty to menopause, is determined by 1) the maximal number of primordial follicles initially formed in the ovary, 2) the rate of primordial follicle activation and 3) the rate of primordial follicle death [12, 13]. Several different factors have been shown to prematurely deplete primordial follicle numbers, including cancer treatments [14–17], environmental toxicants [18–21], infection [22] and inflammation [23], with implications for fertility, depending on the extent of primordial follicle loss.

In the clinical setting, serum AMH is often used as a non-invasive surrogate marker to monitor the size of the ovarian reserve during maternal ageing, as well as before and after cancer treatment (reviewed [16, 24]). However, serum AMH levels do not inform on absolute primordial follicle number, and cannot be used to precisely assess the degree by which exogenous insults reduce primordial follicle number in women. To overcome this limitation, rodents have become widely used as experimental models to define the potential ovo-toxic effect of a variety of existing and new medicines or treatments [25–27], and because of this, refining methods of follicle quantification has become increasingly important. Equally, it is essential that researchers have a thorough understanding of the strengths and weakness of the available counting approaches to ensure appropriate interpretation of results. A survey of the literature reveals that histomorphometric evaluation of follicle numbers in mice presents with immense variability in terms of absolute numbers, likely due to a number of variables like strain, age and treatment type and dose, but also resulting from technical differences in the methods of counting employed [28].

The two most widely reported techniques of ovarian reserve quantification are stereology, using the fractionator optical dissector method, and direct follicle counts (also referred to as follicle estimates) [4, 18, 19, 29–33]. For stereology, objects, in this case follicles, are counted within a known fraction of the total ovary using an optical dissector, which is three-dimensional counting frame, for counting objects in a thick tissue section [34]. The raw count is then multiplied by the inverse of the sampling fractions to determine total follicle number. Stereology accounts for the three-dimensional structure of the object of interest by defining key parameters, which can be then used to systematically identify the structure based on two-dimensional images [35]. As no particular orientation is preferred, the sampling is also isotropic, with the generation of the counting grid being randomized. Based on these principles, stereology is considered to be the gold-standard for cell counting, as with proper sampling parameters, the results derived from this technique are an unbiased and accurate estimate of primordial follicle numbers [4, 35]. This method requires specialist equipment, including a microscope with a motorized stage driven by stereological software. In addition, ovarian samples should be fixed in Bouin’s, and embedded in glycomethacrylate resin to enable the preparation of thick sections (e.g. 20 μm) using a microtome fitted with a glass knife [4, 36]. These parameters are designed to control for shrinkage while optimally retaining three-dimensional morphological detail [37]. The counting process is manually controlled and extremely labor intensive. Thus, despite the accuracy and sensitivity afforded, the associated costs, histological prerequisites, equipment and expertise required, make stereology prohibitive for many laboratories and thus this technique is less widely used.

Conversely, the direct follicle counts technique is widely reported in studies of ovarian reserve in mice. It involves fixing ovaries, most often in formalin, though a variety of different fixatives have been reported, followed by paraffin embedding and serial sectioning at a thickness of 4–6 μm. Follicles are then systematically counted in sections at a regular interval, from a random start, and the number of follicles counted is then multiplied by the inverse of the sampling fraction to obtain the total follicle estimate. If it is considered important for the absolute number of follicles to be accurately calculated, as opposed to relative differences between control and treatment groups for example, additional correction factors can be applied to these raw numbers [28, 38]. Correction factors attempt to account for the assumption that larger follicles are over counted because they appear in more sections, with the inverse true for smaller follicles, but in practice they are only sporadically used. The direct counting method is quick, easy, can be done on archived tissue prepared using standard histological techniques and requires only a light microscope with standard imaging capabilities. However, it does not account for volume changes caused by histological processing, morphology is not always adequately preserved making follicular identification challenging, and the overall accuracy of absolute values for total follicle numbers obtained is unclear.

Follicle density is another common follicle quantification method described in the literature. This technique encompasses counting follicles in a tissue sample and expressing the counts per tissue area. But, follicle density does not control for uneven follicle distribution within the ovary, or changes in ovarian volume, which occur routinely throughout the luteal cycle. Importantly, even within the same mouse ovary, stereology counts do not correspond with follicle density [39], demonstrating the lack of accuracy and sensitivity of this quantification technique. Future directions in the field may include the expansion and uptake of new techniques. A new report of automated detection uses convolutional neural networks driven by labelled datasets and a sliding window algorithm to select test data to count primordial follicle oocytes [40]. But, the algorithm was only tested in two samples. Some studies outline effective methods for clearing mouse ovaries [41, 42], however, do not demonstrate consistent antibody labelling of both oocytes and granulosa cells, crucial for precise follicle classification and enumeration. More recent advances light sheet microscopy have permitted comprehensive analysis of intact tissues, including the ovary [43, 44], though tissue culture is often required and protocols are not optimized for adult animals [45]. Furthermore, an inherent limitation of any automated counting method for ovarian follicles, is the need to visually assess follicle or oocyte health. For now, counting follicles in histological sections remains the most accurate and widely reported means of follicle enumeration.

Whichever approach is utilised, it must be sufficiently sensitive and reproducible to answer the experimental question under investigation, as well as technically and practically feasible. In 2004, Myers et al. compared mouse primordial follicle numbers obtained using two different stereological approaches and reported that physical and optical disectors produce similar results, though it was noted that the optical disector method has the advantage of time efficiency (4). In this study, we sought to expand on this early work by comparing follicle numbers obtained using the fractionator/optical disector technique, as the gold-standard baseline, with those obtained by direct counting. We also investigated the impact of fixative and embedding material on the data obtained, and conclusions drawn, using each method. A well characterised model of primordial follicle depletion was used, in which mice were treated with saline, or a 75 mg/kg/body weight dose of cyclophosphamide, which allowed us to evaluate which method enabled the detection of primordial follicle depletion.

## Results

### Estimation of follicle numbers in saline and cyclophosphamide treated ovaries by stereology and direct counts using standard parameters

**Fig. 1:**
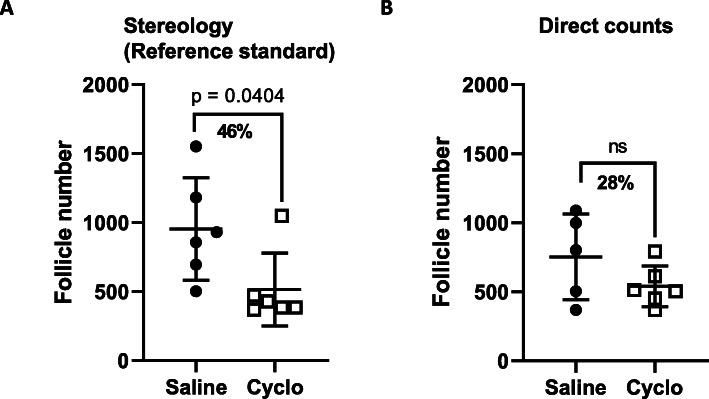
Follicle numbers in saline and cyclophosphamide treated ovaries by stereology and direct counts. **a** For stereology, tissue was fixed with Bouin’s, embedded in resin and 20 μm sections prepared. Every 3rd section was counted and follicle numbers calculated by multiplying the raw counts by the sampling fractions. Cyclophosphamide depletes the number of primordial follicles by 46%. **b** For direct counts, tissue was fixed with formalin, embedded in paraffin and 5 μm sections prepared. Every 9th section was counted and follicle estimates calculated by multiplying the raw counts by the sampling fraction. Cyclophosphamide depletes the number of primordial follicles by 28%. Data are represented as mean ± SEM; unpaired t-test; *n* = 5–6/group; ns = non-significant

We first used fractionator optical dissector technique to determine healthy primordial follicle numbers in Bouin’s fixed, resin embedded, 20 μm sections from saline and cyclophosphamide treated mice (Group 1). This methodology and histological preparation is proposed to provide the most accurate data (Myers, et al. 2004) and therefore formed the baseline for our comparative analyses. The number of primordial follicles per ovary in saline treated mice was 953 ± 253, whereas the number of primordial follicles per ovary in cyclophosphamide treated mice was 407 ± 19 (*p* = 0.0404) (Fig. 1). This represented a 46% reduction in primordial follicle numbers. We next evaluated follicle numbers using direct counts of formalin fixed paraffin embedded, 5 μm sections from the contralateral ovaries in same group of saline and cyclophosphamide treated mice (Group 3). This is representative of a widely used strategy for follicle counting. Using this method, the number of primordial follicles per ovary in saline treated mice was 752 ± 138 (*n* = 5), similar to stereology, but the number of primordial follicles per ovary in cyclophosphamide treated mice was 540 ± 60 (*n* = 6). This represented a 28% reduction in primordial follicle number, and the difference, when compared to saline, was not statistically significant (*p* = 0.1683) (Fig. 1). Thus, the direct count method was unable to detect follicle depletion to the same degree of sensitivity as stereology when performed using recommended histological parameters.

### Estimation of follicle numbers in saline and cyclophosphamide treated ovaries by stereology using different fixation and embedding materials

**Fig. 2:**
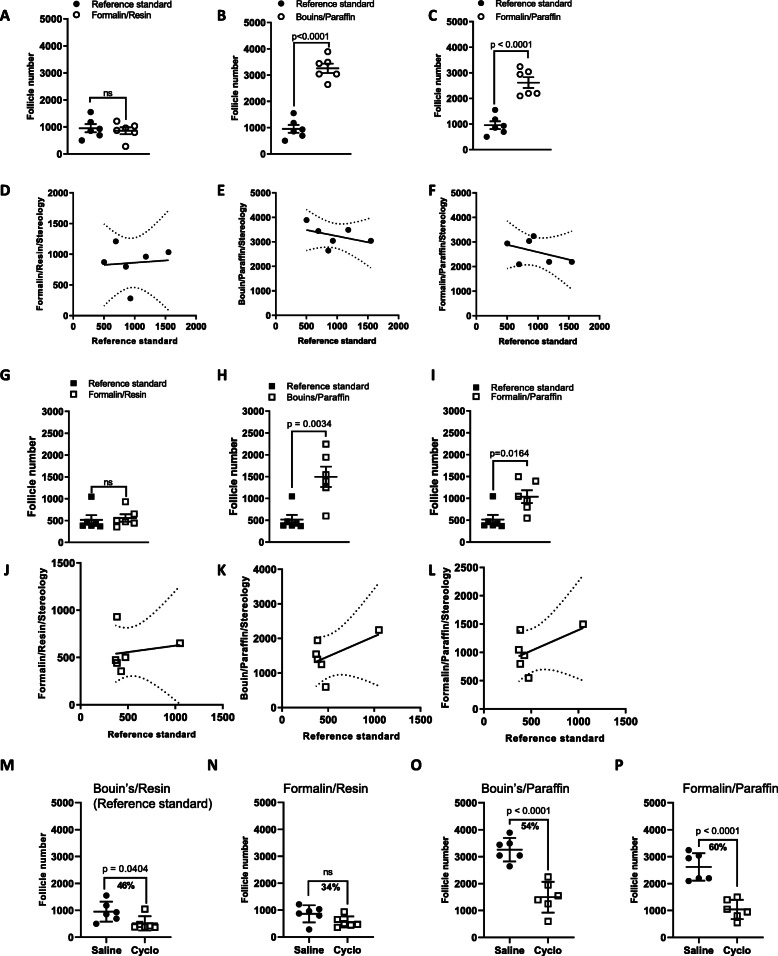
Comparison of follicle numbers obtained by stereology using different fixative and embedding combinations. Follicle numbers in ovaries from mice treated with saline (**a**-**f**) or cyclophosphamide (**g**-**l**), were determined using stereology. Ovaries were fixed in Bouin’s or formalin and embedded in paraffin or resin and sectioned at 5 μm or 20 μm. Follicle numbers for each fixative and embedding material combination were compared to the Reference standard method of Bouin’s fixation, resin embedding and 20 μm sections. Differences in follicle number between ovaries from saline or cyclophosphamide treated mice were also analysed when stereology was applied to tissues fixed and embedded in different combinations (**m**-**p**). The analysis shown in M is the same as Fig. 1a, and is included here to enhance interpretation of (**o**-**p**). Data are represented as mean ± SEM; standard t-test (**a-c**, **g**, **h**, **i**, **m-p**) or correlation plots carried out using linear regression analysis (**d-f**, **j-l**); *n* = 5–6/group; ns = non-significant

Bouin’s fixed and resin-embedded ovaries, with thick sectioning, are considered to be best practice for the histological preparation of tissue prior to stereological counting using the optical disector technique. However, it is unclear if in practice, different fixation, embedding material and section thickness could give comparable results. Therefore, stereological follicle counts using the best practice histological methods, referred to as the “Reference standard”, were compared with counts obtained from tissues prepared using different fixative, embedding material and section thickness combinations (Fig. 2).

Healthy primordial follicle numbers in formalin fixed/resin embedded tissue were not significantly different to Bouin’s fixation (Fig. 2a, g). However, follicle numbers in tissue embedded with paraffin were significantly higher than resin, irrespective of fixative (Fig. 2b, c, h, j). The number of follicles were correlated for each fixative/embedding material combination with the Reference standard to assess if there was a strong relationship between groups. No correlation was found with the numbers predicted using the best-practice methods for saline treated ovaries (Fig. 2d, e, f). However, for cyclophosphamide treated ovaries, when the number of follicles were correlated for each fixative/embedding material combination, a weak correlation (*R*^*2*^ = 0.2870) between Bouin’s fixed/paraffin embedded and the Reference standard was noted (Fig. 2 k); whereas with formalin fixed/paraffin embedding, the correlation was weaker (*R*^*2*^ = 0.2916) (Fig. 2l). There was no correlation between the number of follicles in formalin fixed/resin embedded with the Reference standard (Fig. 2j).

Notably, despite differences in absolute follicle numbers between paraffin and resin embedded samples, stereological principals applied to paraffin embedded formalin or Bouin’s fixed tissues, sectioned at 5 μm, were still useful for detecting follicle depletion by cyclophosphamide, similar to the Reference standard (Fig. 2m, o, p). However, formalin fixation appeared to reduce the sensitivity of analyses in resin embedded tissues (Fig. 2n).

### Estimation of follicle numbers in saline and cyclophosphamide treated ovaries prepared using different fixation and embedding materials by direct count

**Fig. 3:**
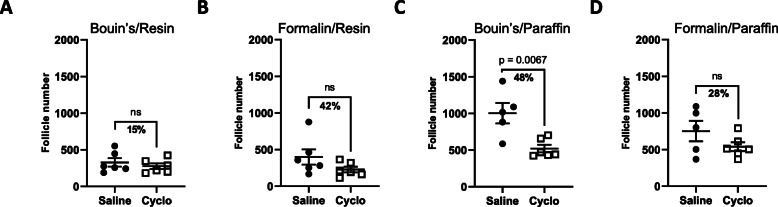
Comparison of follicle numbers obtained by direct counts using different fixative and embedding combinations. Follicle numbers in ovaries from mice treated with saline or cyclophosphamide were determined using direct counts. Ovaries were fixed in Bouin’s (**a**, **c**) or formalin (**b**, **d**) and embedded in paraffin (**c**, **d**) or resin (**a**, **b**) and sectioned at 5 μm (**c**, **d**) or 20 μm (**a**, **b**). The analysis shown in D is the same as Fig. 1b, and is included here to enhance interpretation of (**a**-**c)**. Data are represented as mean ± SEM; standard t-test; *n =* 5–6/group); ns = non-significant

In our first analysis, we observed that direct counts using formalin fixed paraffin embedded 5 μm tissue sections failed to detect a statistically significant difference in follicle number between saline and cyclophosphamide treated ovaries (Figs. 1a, b, and 3a). Thus, we decided to evaluate the impact of different fixative and embedding combinations on this outcome. Interestingly, in contrast to formalin fixation, we found that a significant depletion of primordial follicles caused by cyclophosphamide was detected in Bouin’s fixed paraffin embedded tissue sections, by direct counts. (Fig. 3c). Additionally, these results were very similar to those obtained for the gold standard stereology (Fig. 1a). However, no significant differences were observed between saline and cyclophosphamide treated ovaries following direct counts in resin embedded material (Fig. 3a-d).

### Comparison of follicle numbers obtained using stereology and follicle estimates for each fixative and embedding material combination

**Fig. 4:**
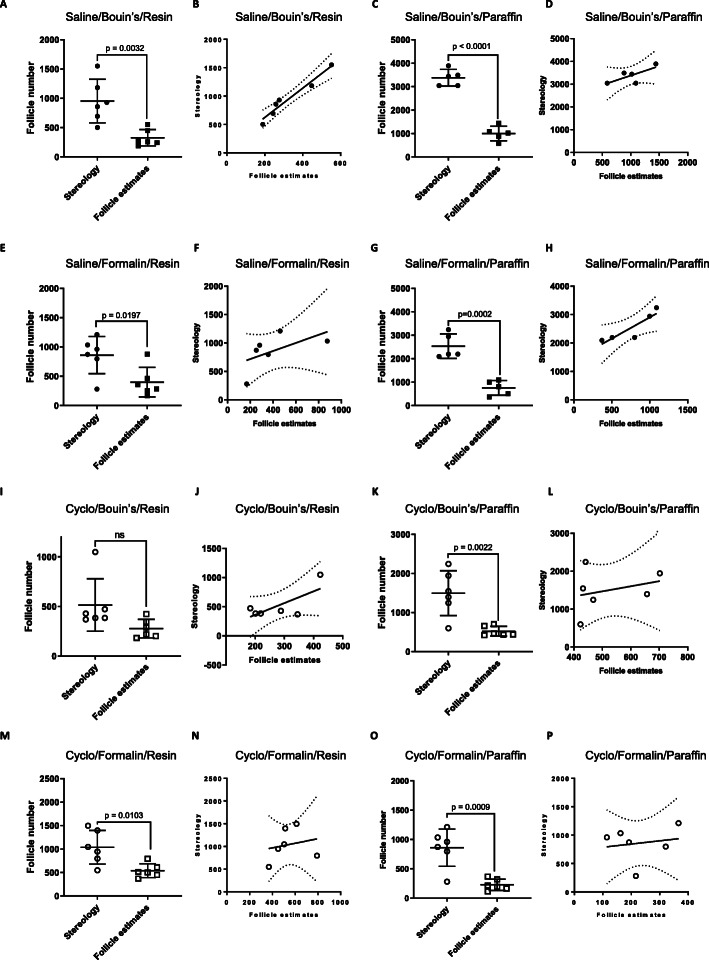
Comparison of follicle numbers obtained by stereology and direct counts in saline and cyclophosphamide treated ovaries, following different combinations of fixation and embedding material. Follicle numbers in ovaries from mice treated with saline (**a**-**h**) or cyclophosphamide (**i**-**p**) were compared following stereology or direct counts. Ovaries were fixed in Bouin’s (**a-d**, **i-l**) or formalin (**e-h**, **m-p**) and embedded in paraffin and sectioned at 5 μm (**c**, **d**, **g**, **h**, **k**, **l**, **o**, **p**) or resin and sectioned at 20 μm (**a**, **b**, **e, f**, **i**, **j**, **m**, **n**). Data are represented as mean ± SEM; standard t-test (A, B, C, G, H, J) or correlation plots carried out using linear regression analysis (**d-f**, **j-l**); *n =* 5–6/group; (**d**, **e**, **f**, **i**, **k**, **l**); *n =* 5–6/group); ns = non-significant

We next directly compared follicle numbers obtained using stereology with those obtained by direct follicle counts for each fixative/embedding material combination. To do this, each sample was analysed by both stereology and direct counts. Overall, direct counts resulted in primordial follicle numbers that were significantly lower than those obtained by stereology, irrespective of fixation and embedding material (Fig. 4a, c, e, g, i, k, m, n). For saline treated ovaries, there was a positive correlation between follicle numbers using Bouin’s fixed/resin embedded material (*R*^*2*^ = 0.9476) and a weak correlation between Bouin’s fixed/paraffin embedded material (*R*^*2*^ = 0.5314) (Fig. 4b, d). There was also positive correlation between formalin fixed/paraffin embedded material (*R*^*2*^ = 0.0.7872) (Fig. 4h) but no correlation between follicle numbers derived using stereology or follicle estimates in tissue fixed in formalin and embedded in resin (Fig. 4f). There was no correlation between the follicle numbers derived using stereology or follicle estimates in tissue fixed with formalin and embedded in either paraffin or resin (Fig. 4f, h). There was a weak correlation (*R*^*2*^ = 0.5096) between numbers of follicles using Bouin’s fixed/resin embedded material. (Fig. 4j); There was no correlation between the numbers of follicles using the two counting techniques for cyclophosphamide treated ovaries for the other fixative/embedding material combinations (Fig. 4l, n, p).

### Morphology of primordial follicles in histological sections used for counting

**Fig. 5:**
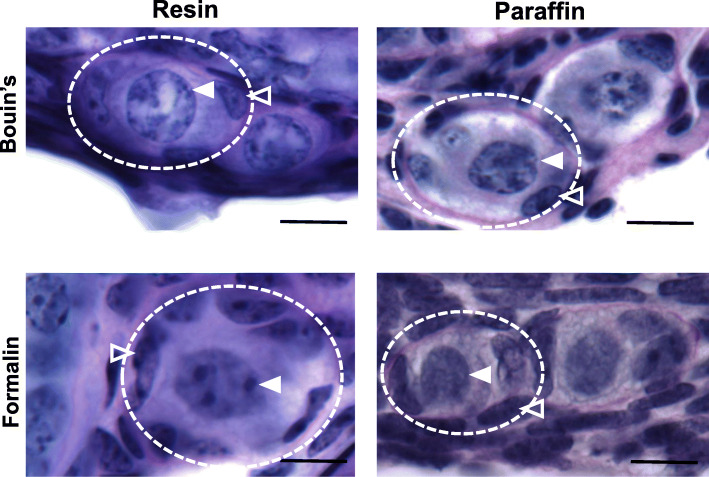
Primordial follicle morphology in ovarian tissue prepared using different fixative and embedding combinations. Primordial follicle structure (dotted lines) following fixation with either Bouin’s solution or formalin and embedded in resin or paraffin. Filled arrow heads show the nucleus of the oocyte. Outlined arrow heads point to one squamous granulosa cell that surrounds the oocyte. Scale bars = 10 μm

One possible explanation for the variation in total follicle numbers obtained using different methods could be because the preservation of follicular morphology is impacted by the fixative and embedding materials used, affecting the ability of researchers to consistently identify primordial follicles. This may be especially problematic for those with less experience. We found follicle morphology to be well preserved in samples fixed with Bouin’s and formalin (Fig. 5). However, thick sections, made possible by resin embedding, enabled greater certainty with regards to follicle classification as the entire follicle could be observed in 3 dimensions.

## Discussion

Accurate and reproducible estimation of primordial follicle numbers is essential for understanding how different endogenous and exogenous factors impact the ovary. In this study, cyclophosphamide-mediated follicle depletion was used to evaluate healthy primordial follicle numbers, and relative differences between control and treatment groups, using two methods of follicle quantification. The objectives were to determine if direct counts are as reliable as the gold-standard stereological method for detecting differences between control and treatment groups, and if different fixatives and embedding materials impact on follicle numbers and experimental outcomes.

In this study we found that, unlike stereology, the direct count method was unable to detect statistically significant follicle depletion in our cyclophosphamide treated samples when performed using the most commonly applied histological parameters for each method (i.e. Stereology: bouin’s fixation/resin embedding/20 μm thick sections; Direct counts; formalin fixation/paraffin embedding/5 μm thick sections). Considerable variability was observed in follicle numbers after cyclophosphamide depletion when direct counts were used, which may underlie this outcome. This variability may be intrinsic to the direct counting method, as it was not observed when tissue sections from the same ovaries were counted using stereology. Despite this limitation, direct counts could still be appropriate when large differences in follicle numbers are expected between groups. Additionally, the power to detect smaller differences in follicle numbers using direct counts could be improved by increasing sample size. It should be noted, however, that we chose 5–6 ovaries per group for these analyses to reflect sample sizes commonly reported. Published studies of follicle numbers, including our own, rarely use more than 6 ovaries per group, and many studies draw conclusions based on only 3 samples. Based on the data presented here, it is very important that lack of a statistically significant difference in follicle numbers is not interpreted as definitive evidence of a lack of biological effect.

One of the limitations of the above analysis is that the primary comparison of follicle number using stereology and direct counts was done on different ovaries (although they were from the same mouse). This was an experimental necessity, because as described, best practice for each method requires ovaries to be prepared using different histological parameters.

Researchers do not always have control over how samples are prepared, nor does everyone have access to stereological equipment, therefore we also sought to investigate the impact of different fixatives and embedding materials on follicle numbers for each counting method. Importantly, we found that absolute follicle numbers, and relative differences between control and treated ovaries, were very similar in ovaries fixed in Bouin’s, embedded in paraffin, sectioned at 5 μm and evaluated using direct counts, compared to data obtained using the best practice stereology method. While it is not certain why Bouin’s produces better results than formalin when direct counts are used, these findings suggest that this histological preparation and counting method combination is a good alternative to stereology. Indeed, Bouin’s fixative is cheap and readily available, and direct counts do not require specialist resin embedding or the preparation of thick sections, which are needed for stereology. Our findings also show that follicle structure is better preserved using Bouin’s fixative, compared with formalin, making identification of the primordial follicle easier. Direct counts can also be performed using a standard microscope with a camera attached and have the additional benefit of being considerably more time efficient than stereology.

Sampling fractions for direct counts are an additional variable; fluctuating in the literature, between every 3rd to every 10th tissue section [46]. As paraffin sections are thin, sampling every 9th section ensures unnecessary counting and oversampling when assessing large numbers of animals [27].

The data also suggest that when performing stereology on resin embedded thick sections, Bouin’s and formalin can be used interchangeably, without impacting the follicle numbers obtained. This is an important finding because it increases the range of tissues that can be assessed using stereology. For example, this observation indicates that historical tissues, fixed in formalin, may be reliably assessed using stereology, as Bouin’s fixation is not a necessity. Indeed, formalin is widely regarded as the superior, or routine fixative of choice [47], due to the versatility of being able to use intervening sections for other histological techniques. Interestingly, although performing stereology on Bouin’s or formalin fixed paraffin sections over-inflates absolute numbers compared to gold standard thick resin sections, this approach did reliably detect statistically significant differences in follicles numbers between controls and cyclophosphamide treated ovaries. Therefore, stereological assessment of tissues routinely prepared in paraffin and sectioned at 5 μm, may be an adequate alternative for some experiments in which identifying treatment effects, rather than absolute follicles numbers, is the primary objective. This is a surprising finding because the stereological fractionator method used in this study employed an optical probe that is designed for counting objects by focusing down through thick tissue sections [4, 36]. None-the-less, in practice the method appears to behave satisfactorily using thin sections.

## Conclusions

The major finding of this study is that direct counts of primordial follicles in Bouin’s, but not formalin-fixed, paraffin embedded ovarian sections are similar to those obtained by the established gold standard stereological method for follicle counting, which utilizes 20 μm thick resin sections. It is clear from the data presented here, that histological preparation of ovarian tissue and counting methodology plays a significant role in the number of primordial follicles estimated and that this must be considered when designing the experiment and interpreting the biological significance of the results obtained. In particular, when direct counts are performed using formalin fixed tissue on small sample sizes, lack of statistically significant differences between groups should not be viewed as definitive evidence of an absence of biological effect.

## Methods

### Animals

Female 8-week-old (reproductively young) C57BL/6 mice were housed in a temperature controlled high barrier facility (Monash University ARL), with free access to food and water, under a 12-h light-dark cycle. All animal procedures and experiments were performed in accordance with the NHMRC Australian Code of Practice for the Care and Use of Animals and approved by the Monash Animal Research Platform Animal Ethics Committee.

### Experimental design

Mice (*n* = 5–6/age/treatment) were weighed prior to a single subcutaneous injection of 75 mg/kg/body weight of cyclophosphamide (Sigma #C0768-5G), or saline vehicle control. This dose has been shown to cause an approximate 50% reduction in primordial follicle pool, and was not reported to cause morbidity or mortality in mice [48]. Mice were humanely euthanized 48 h following injection. Ovaries were harvested and the left and right randomly allocated to a fixation solution; one from each animal was fixed in 10% (v/v) neutral buffered formalin solution (#ANBFC, Australian Biostain) for 24 h, and the other fixed in Bouin’s solution (picric acid 0.9% w/v, formaldehyde 9% v/v, acetic acid 5% w/v, #HT10132, Sigma-Aldrich) for 24 h. Tissue was then either embedded in glycomethacrylate resin (GMA, Technovit 8100, #64709003, Emgrid Australia) and serially sectioned at 20 μm with a RM2165 microtome (Leica Microsystems), or embedded in paraffin and serially sectioned at 5 μm with a MicroTec Cut 4060 paraffin microtome. All tissues were stained with periodic acid-Schiff and haematoxylin.

This resulted in 4 groups for comparison (Table 1). Group 1 ovaries were fixed in Bouin’s, embedded in hydroxyethyl methacrylate resin, and sectioned at 20 μm, which is the standard protocol for the preparation of samples for stereology. Group 2 ovaries were the same as Group 1, except ovaries were fixed in formalin. Group 3 ovaries were fixed in formalin, embedded in paraffin, and sectioned at 5 μm, which is the standard protocol for the preparation of samples for follicle estimation by direct counts. Group 4 ovaries were the same as Group 3, except ovaries were fixed in Bouin’s. Follicle numbers were then estimated in these samples using stereology and/or direct counting methods. Two different investigators performed ovarian follicle analysis. Equal numbers of tissues from each treatment group were assigned to each investigator, then each ovary was assigned a code to de-identify the tissues and ensure the investigators counted whilst blinded.

**Table 1:** Experimental groups showing treatment, fixative, embedding material and counting method

Group	n ovaries	Treatment	Fixative	Embedding material	Counting method
1	6	Saline	Bouin’s	Resin	Stereology/Direct counts
	6	Cyclophosphamide	Bouin’s	Resin	Stereology/Direct counts
2	6	Saline	Formalin	Resin	Stereology/Direct counts
	6	Cyclophosphamide	Formalin	Resin	Stereology/Direct counts
3	5	Saline	Formalin	Paraffin	Stereology/Direct counts
	6	Cyclophosphamide	Formalin	Paraffin	Stereology/Direct counts
4	6	Saline	Bouin’s	Paraffin	Stereology/Direct counts
	6	Cyclophosphamide	Bouin’s	Paraffin	Stereology/Direct counts

### Morphological classification of follicles

This study focused on healthy primordial follicles for two reasons. Firstly, primordial follicles comprise the ovarian reserve and are the focus of the majority of studies evaluating the impact of various insults on the ovary. Secondly, sample preparation and software parameters for stereology have been optimized for small primordial follicles, with larger follicles requiring different sampling parameters. Healthy primordial follicles were defined by the presence of an intact oocyte surrounded by a singular (partial or complete) layer of squamous granulosa cells [5].

### Stereology

Primordial follicle counts were performed as previously published [4]. Stereology was performed using 100x/1.3NA oil immersion objective on a brightfield upright Olympus BX61 microscope with Marhzhauser motorised stage mounted with a DP73 colour camera (Olympus) to generate real-time images in colour. Counts conducted using VisioPharm Stereology software (v7.01.3105 (2017.2)). This method involves the sequential digital overlay of software-generated optical disector counting frames (3D probes) on stained sections using systematic random sampling rules [49]. Every 3rd (resin) or 9th (paraffin) section throughout each entire ovary was evaluated and raw numbers for primordial follicles (Q-) were quantified. The total follicle numbers were determined by multiplying the raw counts by three sampling fractions (1/f1, 1/f2, 1f3). The sampling fractions were f1 = every nth section (3rd for resin, 9th for paraffin), f2 = Counting frame/distance between grids (2250/10,000) and f3 = optical sectioning of the tissue thickness (10/20 for resin, 4/5 for paraffin). Only follicles in which the oocyte nucleus was visible were included in the raw counts. These parameters were designed so that approximately 100 objects were counted in saline treated ovaries.

### Direct follicle counts

Slides were scanned at 20x using an Aperio Digital Pathology Slide Scanner (Leica Biosystems). The Aperio Imagescope program was used to quantify every primordial follicle in every 9th section to obtain raw counts of oocytes sampled (Q-).

The total follicle number was determined by multiplying the raw counts by 9 to correct for the sections not counted. This method of follicle estimates was used to quantify follicles in all tissue fixed in formalin or Bouin’s and embedded in resin or paraffin, and the results were compared to numbers obtained using stereology.

### Statistical analyses

All data was analysed using Graphpad Prism 8 (Version 8.0.2). For each data set, outliers were determined using the Grubb’s method, whereby any outlier is detected from a Gaussian distribution of data. All data are presented as mean ± SEM and *p*-values for each graph are specified following unpaired t-tests. Graphs showing correlations between counting methods for each processing technique were carried out using linear regression analysis with 95% confidence bands of the best-fit line showing.

## Data Availability

The datasets used and/or analysed during the current study are available from the corresponding author on reasonable request.
